# Learning in Autism: Implicitly Superb

**DOI:** 10.1371/journal.pone.0011731

**Published:** 2010-07-22

**Authors:** Dezso Nemeth, Karolina Janacsek, Virag Balogh, Zsuzsa Londe, Robert Mingesz, Marta Fazekas, Szilvia Jambori, Izabella Danyi, Agnes Vetro

**Affiliations:** 1 Institute of Psychology, University of Szeged, Szeged, Hungary; 2 American Language Institute, University of Southern California, Los Angeles, California, United States of America; 3 Department of Experimental Physics, University of Szeged, Szeged, Hungary; 4 Department of Child and Adolescent Psychiatry, University of Szeged, Szeged, Hungary; 5 Graduate School of Educational Sciences, University of Szeged, Szeged, Hungary; University of Regensburg, Germany

## Abstract

**Background:**

Although autistic people have shown impairments in various learning and memory tasks, recent studies have reported mixed findings concerning implicit learning in ASD. Implicit skill learning, with its unconscious and statistical properties, underlies not only motor but also cognitive and social skills, and it therefore plays an important role from infancy to old age.

**Methodology/Principal Findings:**

We investigated probabilistic implicit sequence learning and its consolidation in Autism Spectrum Disorder (ASD). Three groups of children participated: thirteen with high-functioning ASD, 14 age-matched controls, and 13 IQ-matched controls. All were tested on the Alternating Serial Reaction Time Task (ASRT), making it possible to separate general skill learning from sequence-specific learning. The ASRT task was repeated after 16 hours. We found that control and ASD children showed similar sequence-specific and general skill learning in the learning phase. Consolidation of skill learning and sequence-specific learning were also intact in the ASD compared to the control groups.

**Conclusions/Significance:**

These results suggest that autistic children can use the effects/results of implicit learning not only for a short period, but also for a longer stretch of time. Using these findings, therapists can design more effective educational and rehabilitation programs.

## Introduction

Implicit learning is defined as the acquisition of information or motor skill without conscious access to what was learned or even to the fact that learning occurred [1], [2]. Autism Spectrum Disorder (ASD) is characterized by social, communicative and motor impairments [3]. The semantic and episodic memories of people with autism have often been studied, but neurocognitive studies of procedural learning and implicit cognition have received less attention. The extent of learning abilities of ASD individuals is debated [4]. In the present study, we examined implicit motor skill learning in ASD to probe the functional integrity of this type of fundamental learning mechanism.

Most models of motor skill learning [5], [6], [7], [8], [9] emphasize the role of the basal ganglia and the cerebellum, while the role of the hippocampus in this process remains inconclusive [10], [11]. Neuropsychological studies have shown that sequence learning is impaired in people with Huntington's and Parkinson's diseases [12], demonstrating the impact of striatal dysfunction on this type of perceptual-motor learning. Functional brain imaging studies also show the involvement of the cerebellum, striatum and motor cortices in implicit sequence learning tasks including the Serial Reaction Time (SRT) and the Alternating Serial Reaction Time (ASRT) tasks [13], [14], [15]. In addition, Muller et al. [16] reported that autistic individuals showed abnormal fMRI activity patterns in premotor cortex as well as greater individual variability in the activation maps.

Previous studies showed mixed results regarding implicit sequence learning of autistic people. Mostofsky and colleagues [17] found impaired sequence learning when testing autistic children. They used the SRT task, developed by Nissen and Bullemer [18], in which participants were instructed to respond as quickly and as accurately as possible to the location of a stimulus that was presented at one of four possible locations on the monitor in a series of trials. Unknown to the participants, the locations of stimuli follow a predefined sequence, and participants typically become faster at responding to the locations predicted by the sequence compared to random trials. Mostofsky et al. [17] tested a 10-trial fixed sequence repeated 8 times in a block, across a total of 5 blocks using a longer 1500 ms interval, instead of the customary 120–300 ms response-to-stimulus interval used in SRT tasks (e.g., [18], [19]). Gordon and Stark [20] tested sequence learning in autistic participants in two tasks, one with an 8-element, and the other a 4-element fixed sequence. Their results revealed marginal learning with the 8-element fixed sequence task and significant learning with the 4-element task. As in Mostofsky et al. [17], this study used an unusually long response to stimulus interval (RSI) of 500 ms.

Four issues arise with the two studies above: 1) With a fixed-sequence series the possibility of an explicit strategy arises, because it is easier to become aware of the sequence, since the same sequence is presented repeatedly. 2) Both in the 10- and 8-element sequences the frequency of the elements was not balanced. Some elements could have occurred more frequently than others, which could increase the possibility of pattern recognition of the sequence, making the learning process explicit rather than implicit. In addition, it is possible that the learning observed was due at least partly to learning the relative frequencies of individual events rather than of sequences of events. 3) The long RSI values in the above studies could also contribute to developing an explicit strategy. Research has suggested that the longer the RSI, the more probable that explicit strategies are used [21], [22], [23]. 4) In the various neuropsychological and neurodevelopmental disorders in which IQ is involved, it has been found that explicit learning is correlated with IQ, while implicit learning is relatively independent of IQ level [24], [25], [26]. Explicit processes, therefore, suffer more under circumstances with IQ impairment. If learning relies on explicit strategies, then autistic individuals could be learning less than controls due to impairments in explicit rather than implicit learning.

Barnes and colleagues [27] overcame the above limitations by using a 3-element version of the ASRT task [19], which is a modified version of the SRT task. In classical SRT tasks the structure of a sequence is deterministic with the stimuli following a simple repeating pattern as in the series 213412431423, where numbers refer to distinct events. In contrast, in the ASRT task [19], [28] repeating events alternate with random elements. This means that the location of every second stimulus on the screen is determined randomly. If, for instance, the sequence is 123, where the numbers represent locations on the screen, in ASRT the sequence of stimuli will be 1R2R3R1R2R3R…, with R representing a random element. The sequence is thus better hidden than in the classical SRT task and it is also possible to track sequence-specific learning continuously by comparing responses to the random and sequence elements within each testing block. This structure is called a probabilistic second-order (lag-2) dependency [19], [28], because to predict element ‘n’ we need to know element n-2. Barnes et al. [27] used a 120ms RSI, and they found intact learning in Autism compared to a control group matched for age and IQ. The authors suggest that the fronto-striatal-cerebellar functions are spared in autism.

It is possible that Barnes et al. [27] found intact implicit learning because participants were mostly children with Asperger's syndrome, who have better cognitive abilities than children with simple autism. It is also possible that this group found intact implicit learning because they used the ASRT with 3 elements (i.e., 3 possible locations corresponding to 3 possible responses), which could be too easy to detect deficits. However, in a recent study Brown et al. [26] also observed intact implicit sequence learning in a probabilistic SRT task introduced by Schvaneveldt & Gomez [29]. In this task the RSI was 0 ms to reduce the possibility of creating an explicit strategy [21], [22], [26].

To our knowledge, consolidation of implicit or procedural learning has not been studied in autism, although some research has investigated consolidation of episodic and semantic long-term memories [30], [31], [32]. Because some aspects of these domains show impairments in autism, it is important to investigate the implicit consolidation processes as well. When examining consolidation it is essential to know that skill learning occurs not only during practice in the so-called *online* period, but also between-practice during the so- called *offline* phase. The process that occurs during the offline period is referred to as consolidation, which means stabilization of a memory trace after the initial acquisition or even improvement in performance following an offline period [33]. Such consolidation is important in considering the long-term acquisition of skills; even if implicit learning is intact, it is possible that autistic individuals are impaired in consolidation, thus forgetting the skills over the longer term. This might explain the apparent contradiction of intact implicit learning in autistic people even though they are known to be weaker in communicative and social skills [3].

In our study we used the ASRT task to investigate implicit learning and consolidation in autism. The ASRT task allows separation of general skill learning and sequence specific learning during both online and offline periods. General skill learning is reflected in the overall reaction time, whereas sequence-specific learning is reflected in the difference between the reaction time to predictable, sequence events as opposed to less predictable random ones. We also examined the effect of a 16-hour delay on learning performance, to test whether consolidation is intact. The present study goes beyond previous studies [17], [20], [26], [27] in two ways: 1) we used a more difficult 4-element ASRT task with 4 possible locations and 4 corresponding responses, instead of the 3-element version used by Barnes et al. [27], and 2) we investigated the consolidation of implicit learning over a 16-hour period.

## Materials and Methods

### Participants

Thirteen children with ASD, 13 IQ-matched, and 14 age-matched children participated in the experiment. Their characteristics are described in Table 1. The IQ-matched control group differed significantly from the other two groups in mean age (IQ control and ASD: t(24) = 2.25; p = 0.034; IQ and AGE control: t(25) = −2.05, p = 0.51), whereas the mean IQ in the AGE-matched control group was significantly higher than in the ASD (t(25) = −2.12, p = 0.044) and IQ-matched control group (t(25) = −2.12, p = 0.044).

**Table 1:** General data of participants.

	Age		IQ		Sex	ASRT learning
	Mean (SD)	Range	Mean (SD)	Range		
ASD (n = 13)	11.77 (3.14)	7–17	93.15 (20.67)	70–146	11 M/2 F	10/13
IQ-matched control (n = 13)	9.23* (2.59)	8–17	96.54 (17.65)	74–139	13 M	12/13
AGE-matched control (n = 14)	11.57 (3.27)	7–17	109.07* (12.83)	90–138	12 M/2 F	12/14

The IQ-matched control group was significantly younger than the other two groups; and the mean IQ of the AGE-matched control group was the highest (* - p<0.05). The right-most column shows the number of participants in each group who showed significant sequence learning (determined by greater than zero RT difference in high minus low frequency triplets in the last epoch of Session 1).

The children's IQ was measured by the Wechsler Intelligence Scale for Children (WISC, 3rd ed.). All children with ASD were diagnosed using the criteria in the DSM-IV [3], and had received clinical evaluations both according to the Autism Diagnostic Interview (ADI) and the Autism Diagnostic Observation Schedule (ADOS) [34],[35]. The mean score of the ADOS was 3.00 (SD = 1.58) for Communication and 5.67 (SD = 1.87) for Reciprocal Social Interaction domains. The mean score of ADI-R was 10.75 (SD = 4.65) for Reciprocal Social Interaction, 11.25 (SD = 6.15) for Communication and 4.87 (SD = 1.25) for Repetitive Behavior domains. Four of the ASD group members had a diagnosis of Asperger's syndrome. Children with neurological or psychiatric disorders, or IQ of less than 70 were excluded from the experiment. Control groups did not suffer from any developmental, psychiatric or neurological disorders, and did not have sleeping disorders. Parents reported that all children had 7–8 hours of sleep a day. Informed written parental consent and verbal assent of the children were provided, and participants did not receive financial compensation for their participation. Ethics approval was obtained by Psychology Ethics Committee at University of Szeged, Institute of Psychology.

### Procedure

**Figure 1:**
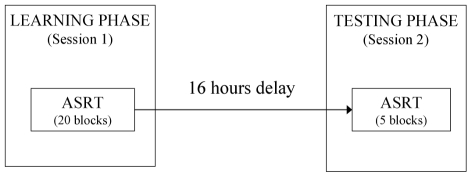
Experiment design. There were two sessions in the experiment: a Learning Phase (Session 1) followed by a Testing Phase (Session 2) after a 16-hour delay.

There were two sessions in the experiment (see Figure 1): a learning phase (Session 1) and a testing phase (Session 2) separated by a 16-hour interval (±2 hours). The first session was in the afternoon (between 2–4 PM), and took approximately 30–35 minutes; the second session was in the morning (between 7–9 AM) and lasted 5–10 minutes.

### Alternating Serial Reaction Time (ASRT) Task

We used a modified version of the original ASRT task [19], in which a stimulus (a dog's head) appeared in one of the four empty circles on the screen and the subject had to press a corresponding key (Y, C, B and M on Hungarian keyboard) when it occurred [36].

Session 1 (the learning phase) consisted of 20 blocks of the ASRT, with 85 key presses in each block - the first five trials were random (for practice and to make it more difficult to discover the pattern explicitly), then the 8-element sequence (i.e., 4 pattern events alternating with 4 randomly determined ones) repeated 10 times. Following Howard et al. [19] each stimulus was presented 120 ms following the previous response (response-to-stimulus interval, RSI). Between blocks, the subjects received feedback about their overall reaction time and accuracy, and then they were given a 10–20 second rest before starting a new block. Session 2 (the testing phase) consisted of 5 blocks of the ASRT, because we only focused on offline changes of previously acquired knowledge [36], [37]. The number of key presses per block and the RSI were the same as Session 1.

There are 6 possible sequences in which each of the four positions occurs once and only once (i.e., 1r2r3r4r, 1r2r4r3r, 1r3r4r2r, 1r3r2r4r, 1r4r2r3r, 1r4r3r2r), and each of these was used approximately equally often across subjects within a group, but the sequence for a given subject was identical during Session 1 and Session 2.

To explore how much explicit knowledge subjects acquired about the task, we administered a short questionnaire (similar to [37]) after the second session. This questionnaire included increasingly specific questions such as “Have you noticed anything special regarding the task? Have you noticed some regularity in the sequence of the stimuli?” The experimenter rated subjects' answers on a 1–5 scale, where 1 was “Nothing noticed” and 5 represented “Total awareness.” None of the subjects reported noticing the sequence either in the ASD, the IQ- or AGE-matched control groups.

### Statistical analysis

As there is a fixed sequence in the ASRT with random elements inserted (e.g. 1 R 2 R 3 R 4 R, when R represents random trials) some triplets or runs of three events occur more frequently than others. For example, for the above example sequence 1×2, 2×3, 3×4, and 4×1 would occur often whereas 1×3 or 4×2 would occur infrequently. Following previous studies, we refer to the former as high-frequency triplets and the latter as low-frequency triplets. Pattern trials are always high-frequency, whereas one-fourth of the random trials are high-frequency by chance. Thus, high-frequency triplets occur 62.5% of the time and low-frequency triplets occur 25% of the time (excluding repetitions, e.g. 333, and trills, e.g. 313). As is typical, we have excluded repetitions and trills from analyses because they usually reveal preexisting response biases and because they are always low frequency for all subjects and hence (unlike the remaining triplets) are not counterbalanced [38]. Earlier results have shown that as people practice the ASRT task, they come to respond more quickly to the high- than low-frequency triplets, revealing sequence-specific learning [19], [37], [38], and participants remain unaware of such learning. In addition, general motor skill learning is revealed in the ASRT task with a decrease in average response speed, irrespective of the triplet types. Thus, we are able to obtain measures of both sequence-specific and general motor skill learning in the ASRT task.

To facilitate data processing, the blocks of ASRT were organized into epochs of five blocks. The first epoch contained blocks 1–5, the second epoch blocks 6–10, etc. [27], [37]. The analyses were performed as in Song et al's [37] and Nemeth et al. [36], [39]. We report both the reaction times (RT) and accuracy data; however, our focus is primarily on RT. For RT we calculated means for correct responses only (eliminating trills and repetitions and RTs that fell more than 3 standard deviations from the mean RT for that subject), separately for trials ending high versus low frequency triplets and for each subject and each epoch. For accuracy, we used the mean percentages of the correct responses.

## Results

### Online learning during session 1

#### Reaction time

**Figure 2:**
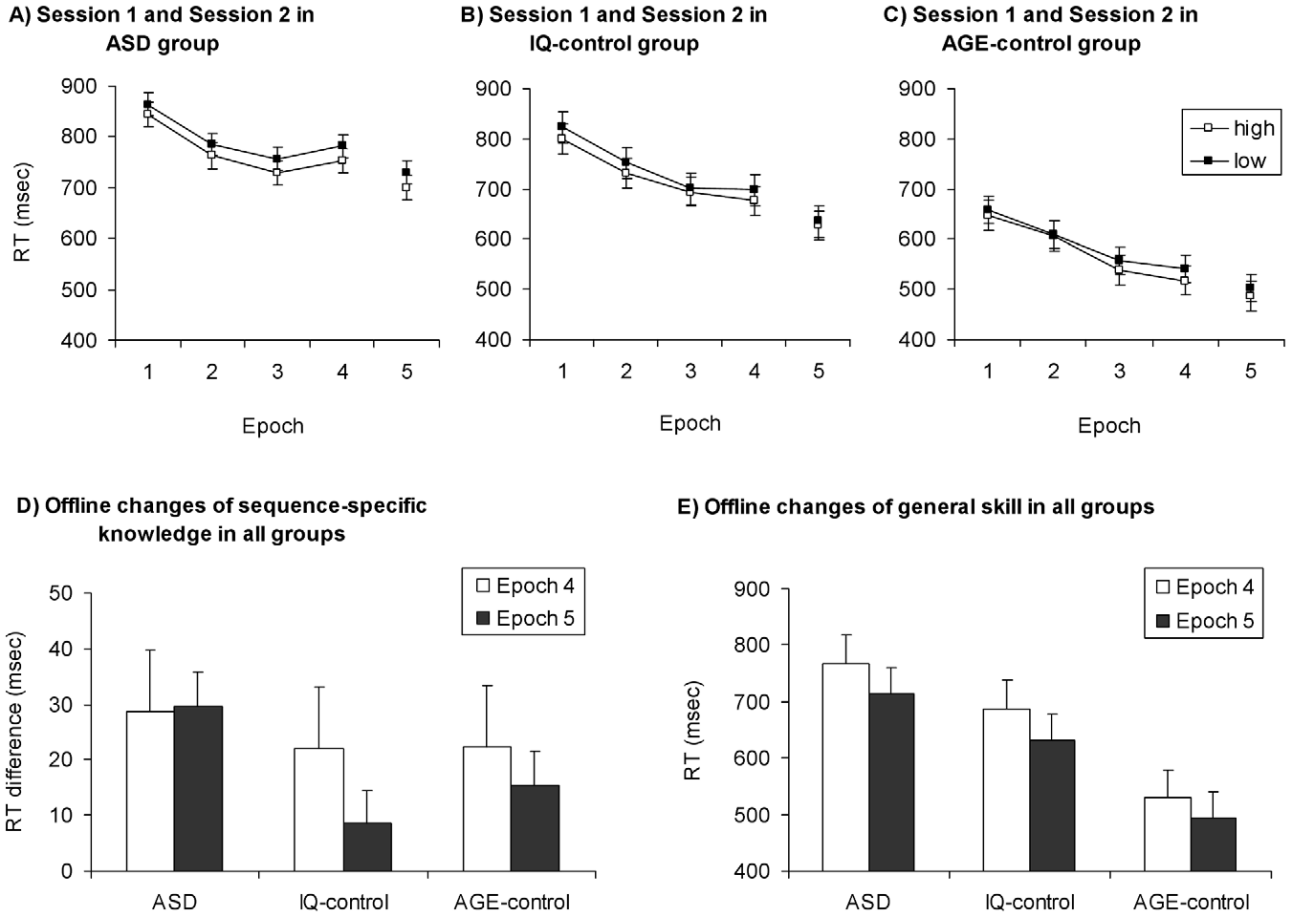
Results of the experiment. RTs of Session 1 (epoch 1–4) and Session 2 (epoch 5) for ASD (A), IQ-matched (B) and AGE-matched (C) control groups. The RT differences between the high (open squares) and low frequency (filled squares) triplets indicate sequence-specific learning, whereas the decrease of reaction time (regardless of triplet type) indicates general skill learning. In Session 1 all groups showed significant sequence-specific and general skill learning. D) Offline changes of sequence-specific knowledge for all groups. The sequence learning effect (SLE) is the RT on low frequency minus RT on high frequency trials; this effect on the last epoch of Session 1 (Epoch 4) does not differ significantly from that of the first epoch of Session 2 (Epoch 5). E) Offline changes of general skill for all groups; there was no difference in overall RT between Epoch 4 and 5 for any group. Error bars indicate SEM.

To investigate learning during Session 1 (learning phase) a mixed design ANOVA was conducted on the first 4 epochs of the RT data shown in Figure 2A–C, with (TRIPLET: high vs. low) and (EPOCH: 1–4) as within-subjects factors, and GROUP (ASD, IQ- and Age-matched control groups) as a between-subjects factor. Thus, sequence-specific learning would be revealed by main effects and/or interactions with TRIPLET.

There was significant sequence-specific learning (indicated by the significant main effect of TRIPLET: F(1,37) = 37.55, MSE = 747.57, p<0.000001, η_p_^2^ = 0.50) such that RT was faster on the high than low frequency triplets. There was also general motor skill learning (shown by the significant main effect of EPOCH: F(3,111) = 14.27, MSE = 15368.84, p<0.000001, η_p_^2^ = 0.28), such that RT decreased across epochs. There were no group differences in learning (no interactions with group were significant; all p's>0.40). The only significant effect regarding Group was the main effect (F(2,37) = 4.58, MSE = 256569.47, p = 0.02, η_p_^2^ = 0.20), reflecting that the Age-matched control group responded faster than both the ASD and IQ-matched control groups (p's<0.04). The ANOVA conducted on transformed data (using the same method as Barnes et al, 2008: low minus high differences in epochs/RT of low frequency triplets) revealed the same results.

Subsequent TRIPLET×EPOCH ANOVAs on the RTs, conducted separately for each group confirmed that each group showed both general skill learning and sequence-specific learning. For the ASD group there was a significant main effect of TRIPLET, F(1,12) = 22.21, MSE = 683.68, p = 0.001, η_p_^2^ = 0.65 and the main effect of EPOCH was F(3,36) = 2.14, MSE = 28145.74, p = 0.11, η_p_^2^ = 0.15. The EPOCH×TRIPLET interaction was not significant, F(3,36) = 0.15, MSE = 987.34, p = 0.93, η_p_^2^ = 0.05. For the IQ-matched control group there were significant main effects of TRIPLET, F(1,12) = 7.29, MSE = 1166.34, p = 0.02, η_p_^2^ = 0.38 and of EPOCH, F(3,36) = 8.40, MSE = 9873.67, p<0.0001, η_p_^2^ = 0.41. The TRIPLET×EPOCH interaction was not significant (F(3,36) = 0.53, MSE = 815.31, p = 0.67, η_p_^2^ = 0.04). For the Age-matched control group the main effects of TRIPLET and EPOCH were also significant (F(1,13) = 13.03, MSE = 420.00, p = 0.003, η_p_^2^ = 0.44; F(3,39) = 10.37, MSE = 8647.24, p<0.0001, η_p_^2^ = 0.50; respectively). The TRIPLET×EPOCH interaction did not reach significance (F(3,39) = 2.21, p = 0.10, η_p_^2^ = 0.15).

#### Accuracy

The same analyses were conducted on accuracy measures. The ANOVA revealed significant sequence-specific learning (indicated by the significant main effect of TRIPLET: F(1,37) = 17.35, MSE = 0.001, p<0.0001, η_p_^2^ = 0.32), such that the accuracy was greater on high than low frequency triplets. The main effect of EPOCH was also significant (F(3,111) = 3.13, MSE = 0.002, p = 0.029, η_p_^2^ = 0.08), such that accuracy decreased across epochs (which reflects falling accuracy for low frequency triplets). There were no group differences in learning (no interactions with group were significant; all p values>0.61). The main effect of Group was not significant (F(2,37) = 1.14, MSE = 0.015, p = 0.33, η_p_^2^ = 0.06), reflecting that all groups responded with similar accuracy rates (ASD group 94%, IQ-matched control 92%, Age-matched control 94%).

Subsequent TRIPLET×EPOCH ANOVAs were conducted separately for each group to confirm the results. For the ASD group there was a significant main effect of TRIPLET, (F(1,12) = 5.37, MSE = 0.001, p = 0.039, η_p_^2^ = 0.31), whereas of the main effect of EPOCH did not reach significance (F(3,36) = 2.21, MSE = 0.002, p = 0.10, η_p_^2^ = 0.15). For the IQ-matched control group there was only a marginally significant main effect of TRIPLET, (F(1,12) = 4.05, MSE = 0.001, p = 0.067, η_p_^2^ = 0.25), whereas the main effect of EPOCH was not significant, (F(3,36) = 0.48, MSE = 0.004, p = 0.70, η_p_^2^ = 0.04). For the AGE-matched control group the main effect of TRIPLET was significant (F(1,13) = 8.36, MSE = 0.001, p = 0.013, η_p_^2^ = 0.39), and the main effect of EPOCH was marginally significant (F(3,39) = 2.83, MSE = 0.001, p = 0.051, η_p_^2^ = 0.18). The TRIPLET×EPOCH interaction was not significant in any group (all p's>0.36).

### Offline changes of sequence-specific knowledge

To define the index for offline sequence-specific learning, we calculated the Sequence Learning Effect (SLE) which is the RT/accuracy difference for the low versus high frequency triplets for the last epoch of Session 1 (Epoch 4). This index shows the magnitude of sequence-specific learning at the end of the first session [27]. Similarly, we calculated this Sequence Learning Effect for the first epoch of Session 2 (Epoch 5). These SLE scores (shown in Figure 2D) were submitted to a mixed design ANOVA with EPOCH (Epoch 4 and 5) as a within-subjects factor and GROUP (ASD, IQ- and Age-matched control groups) as a between-subjects factor. Thus, any offline changes in sequence-specific learning would be revealed by main effects and/or interactions with EPOCH. In the ANOVA on RT difference scores, neither the main effect of EPOCH, nor the EPOCH×GROUP interaction reached significance (F(1,37) = 0.72, MSE = 1157.37, p = 0.40, η_p_^2^ = 0.02; F(2,37) = 0.30, MSE = 1157.37, p = 0.74, η_p_^2^ = 0.02; respectively). The subsequent paired t-tests conducted separately for each group confirmed these results (all p's>0.20). Thus, there was no evidence of offline changes (improvement or deterioration) of sequence-specific knowledge regardless of group.

In the same analysis conducted on the accuracy Sequence Learning Effects (Accuracy on High Frequency minus that on Low Frequency) neither the main effect of EPOCH (F(1,37) = 0.13, MSE = 0.001, p = 0.72, η_p_^2^ = 0.004), nor the EPOCH×GROUP interaction was significant (F(2,37) = 2.24, MSE = 0.001, p = 0.12, η_p_^2^ = 0.11).

### Offline changes of general skills

To examine offline general skill learning we calculated the overall RT/accuracy (combined across triplet types) for the last epoch of Session 1 and the first epoch of Session 2; the greater the RT decrease from Session 1 to Session 2, the larger the offline general skill improvement was. Further, a lack of increase in RT between the two sessions (with a 16-hour time delay between sessions) would signal that the participant's retention of general skill was intact. These overall RTs were used in a mixed design ANOVA with EPOCH (Epoch 4 and 5) as a within-subjects factor and GROUP (ASD, IQ- and Age-matched control groups) as a between subject factor. The ANOVA revealed offline improvement of general skill (shown in Figure 2E) in that the main effect of EPOCH was significant, (F(1,37) = 15.06, MSE = 3012.21, p<0.001, η_p_^2^ = 0.29), reflecting the faster overall RTs for the first epoch in Session 2 compared to those at the end of Session 1. The EPOCH×GROUP interaction was not significant, F(2,37) = 0.28, MSE = 3012.21, p = 0.76, η_p_^2^ = 0.015.

This evidence for offline consolidation of general skill relies on comparing RT on epoch 5 to that on epoch 4, so it is possible that the faster RT on epoch 5 is simply due to learning that occurred during epoch 5 [36]. To rule out this possibility, we conducted the same analysis for Epoch 3 and 4 (within Session 1). Neither the main effect of EPOCH, nor the EPOCH×GROUP interaction was significant (F(1,37) = 0.01, MSE = 7287.32, p = 0.97, η_p_^2^<0.001; F(2,37) = 0.47, MSE = 7287.32, p = 0.63, η_p_^2^ = 0.025). This suggests that the offline effects we observed were not simply due to continued learning.

The results of accuracy analysis also confirmed these findings. When comparing the Epoch 4 and Epoch 5 (across sessions), ANOVA revealed a significant main effect of EPOCH (F(1,37) = 13.82, MSE = 0.001, p = 0.001, η_p_^2^ = 0.27), reflecting an offline increase in overall accuracy (from 92.5% to 95.4%). There was no significant difference among the groups (EPOCH×GROUP interaction: F(2,37) = 1.13, MSE = 0.001, p = 0.33, η_p_^2^ = 0.06). The ANOVA conducted for Epoch 3 and 4 (within Session 1) revealed a trend for a main effect of EPOCH (F(1,37) = 3.01, MSE = 0.001, p = 0.09, η_p_^2^ = 0.075), but with a reverse pattern: they were less accurate in the Epoch 4 compared to the Epoch 3 (93.5% versus 92.5%). The EPOCH×GROUP interaction was not significant (F(2,37) = 0.92, MSE = 0.001, p = 0.41, η_p_^2^ = 0.05).

## Discussion

Our goal was to investigate whether implicit sequence learning and consolidation are impaired in children with ASD. We used a task that allowed us to differentiate between general skill and sequence-specific learning. We found that ASD children showed general skill learning and implicit learning of probabilistic sequences similar to that of two groups of controls, one matched in IQ and the other in age. In addition, the groups did not differ in consolidation; over a 16-hour period between sessions, we observed no forgetting of sequence-specific learning, as well as offline improvements in general skill, with no significant differences among groups. We believe our study to be the first to investigate implicit learning consolidation in autism.

The findings of the online learning (Session 1) are similar to those of Barnes et al. [27] and Brown et al. [26], who also found probabilistic implicit learning to be intact in samples of autistic children. Our results build on these earlier studies in that we show intact learning of a more difficult regularity, in that we used a 4-element ASRT task, instead of the 3-element version in Barnes et al. [27]. Nonetheless, accepting the null hypothesis requires caution. Small sample size and great variability in responses could reduce our ability to detect group differences in learning, however, previous studies with similar findings and similar sample sizes support our conclusions.

Why has the current study and several others found intact implicit sequence learning in this population [26], [27] while others did not [17]? Brown et al. [26] has suggested that explicit strategies could affect the differences in these findings: they reason that such strategies could help in learning deterministic sequences (but not probabilistic ones, since they are more difficult to discover explicitly). This research group also argues that high RSI values could contribute to strategy building: Gordon & Stark [20] and Mostofsky et al. [17] used 500–1500 ms whereas Barnes et al. [27] and Brown et al. [26] used 120 and 0 ms, respectively. Higher RSIs can lead to more explicit strategies and learning [22]. Thus, according to Brown's hypothesis performance is influenced by the use of explicit strategies [26]. Using explicit strategies/explicit processes is highly correlated with IQ levels, whereas implicit processes are not [24], [25], [26]. Brown et al. [26] also reason that ASD individuals are prone to solving tasks explicitly, as shown in several studies (e.g., Theory of Mind performance is mediated explicitly in ASD [40], [41]). Thus, their impairments may be reflecting impaired explicit, not implicit learning. When there is no chance to use explicit strategies, as in our study, or in Barnes' [27] and Brown's [26] the autistic participants are able to reveal their intact implicit learning.

A different hypothesis explaining the contradictory research results can be drawn from Happé & Frith [42] who suggest that ASDs have attentional preference for local over the global context. It is possible that longer RSIs make it even more difficult for ASD participants to engage in global-context processing because the increased time between events makes it difficult to group them. Thus, longer RSIs would put ASD participants at a disadvantage in sequence learning compared to controls. Testing these hypotheses will require more research.

The results of the present study concerning consolidation are similar to those of Song et al. [37] and Nemeth et al. [36]. Like the healthy young and older adults in these earlier studies, our ASD and control groups remembered the sequence between sessions as shown by a lack of decline in Sequence Learning Effect over the 16 hours between sessions. In addition, as had been the case for the adults in these earlier studies, all three groups of children showed offline enhancement of general skill in that they started their second session at a faster response rate than the end of the first session. However, neither study [36], [37] found a sleep effect in the general skill learning or in the sequence-specific learning. This is important because ASD has been highly associated with sleep difficulties [3]. Thus, whether consolidation is intact or defective in autism it is most likely not the result of sleep disturbance. The fact that our ASD children did not show deficits in consolidation, is also consistent with evidence suggesting that sleep may not play a critical role in consolidation of implicit sequence learning [36], [37], [43], [44].

Moreover, our findings draw attention to the fact that children acquire the hidden sequences very fast, as they are sensitive to statistical probabilities already in the first epoch of learning. This early sensitivity may reflect greater neural plasticity and is less typical among adults [36], [37], [45].

In summary, this study found that implicit sequence-specific and general skill learning are unimpaired in participants with ASD, and that consolidation of the learning is intact as well. This suggests that autistic children can use the effects/results of implicit learning not only for a short period, but also for a longer stretch of time. Learning seems to get embedded into the cognitive system, which could play an important role in therapy. Learning in general relies on implicit and explicit processes at the same time. If implicit sequence learning is spared relative to explicit learning in ASD [4], then emphasizing implicit processes could improve real-life learning in ASD. Using these results, therapists can design more effective educational and rehabilitation programs. Our findings suggest that learning mechanisms associated with frontal-striatal-cerebellar anatomy are partly intact in ASD.

## References

[1] Perruchet P, Pacton S (2006). Implicit learning and staticial learning: one phenomenon, two approaches.. Trends in Cognitive Sciences.

[2] Reber AS (1967). Implicit learning of artificial grammars.. Journal of Verbal Learning and Verbal Behavior.

[3] APA (1994). DSM-IV.

[4] Dawson M, Mottron L, Gernsbacher M (2008). Learning in autism..

[5] Hikosaka O, Nakahara H, Rand MK, Sakai K, Lu X (1999). Parallel neural networks for learning sequential procedures.. TINS.

[6] Hikosaka O, Nakamura K, Sakai K, Nakahara H (2002). Central mechanisms of motor skill learning.. Curr Opin Neurobiol.

[7] Doyon J, Bellec P, Amsel R, Penhune V, Monchi O (2009). Contributions of the basal ganglia and functionally related brain structures to motor learning.. Behav Brain Res.

[8] Kincses T, Johansen-Berg H, Tomassini V, Bosnell R, Matthews P (2008). Model-free characterization of brain functional networks for motor sequence learning using fMRI.. Neuroimage.

[9] Robertson EM (2009). From creation to consolidation: A novel framework for memory processing.. PLoS Biology.

[10] Schendan H, Searl M, Melrose R, Stern C (2003). An FMRI study of the role of the medial temporal lobe in implicit and explicit sequence learning.. Neuron.

[11] Albouy G, Sterpenich V, Balteau E, Vandewalle G, Desseilles M (2008). Both the hippocampus and striatum are involved in consolidation of motor sequence memory.. Neuron.

[12] Willingham DB (1997). Systems of memory in the human brain.. Neuron.

[13] Fletcher PC, Zafiris O, Frith CD, Honey RAE, Corlett PR (2005). On the benefits of not trying: brain activity and connectivity reflecting the interactions of explicit and implicit sequence learning.. Cerebral Cortex.

[14] Rauch SL, Whalen PJ, Savage CR, Curran T, Kendrick A (1997). Striatal recruitment during an implicit sequence learning task as measured by functional magnetic resonance imaging.. Human Brain Mapping.

[15] Willingham DB, Salidis J, Gabrieli JD (2002). Direct comparison of neural systems mediating conscious and unconscious skill learning.. Journal of Neurophysiology.

[16] Muller RA, Pierce K, Ambrose JB, Allen G, Courchesne E (2004). Atypical patterns of cerebral motor activation in autism: a funtional magnetic resonace imaging study.. Biol Psychol.

[17] Mostofsky SH, Goldberg MC, Landa RJ, Denckla MB (2000). Evidence for a deficit in procedural learning in children and adolescents with autism: implications for cerebellar contribution.. Journal of the International Neuropsychological Society.

[18] Nissen MJ, Bullemer P (1987). Attentional requirements of learning: Evidence from performance measures.. Cognitive Psychology.

[19] Howard JH, Howard DV (1997). Age differences in implicit learning of higher-order dependencies in serial patterns.. Psychology and Aging.

[20] Gordon B, Stark S (2007). Procedural Learning of a Visual Sequence in Individuals With Autism.. Focus on Autism and Other Developmental Disabilities.

[21] Destrebecqz A, Cleeremans A (2001). Can sequence learning be implicit? New evidence with the process dissociation procedure.. Psychonomic Bulletin & Review.

[22] Destrebecqz A, Cleeremans A, Jiménez L (2003). Temporal effects in sequence learning.. Attention and Implicit Learning.

[23] Jimenez L, Mendez C, Cleeremans A (1996). Measures of awareness and of sequence knowledge.. Psyche: An Interdisciplinary Journal of Research on Consciousness.

[24] Gebauer GF, Mackintosh NJ (2007). Psychometric intelligence dissociates implicit and explicit learning.. Journal of Experimental Psychology: Learning, Memory, and Cognition.

[25] Reber AS, Walkenfeld FF, Hernstadt R (1991). Implicit and explicit learning: Individual differences and IQ.. Journal of Experimental Psychology.

[26] Brown J, Aczel B, Jimenez L, Kaufman SB, Grant KP (2010). Intact implicit learning in autism spectrum conditions.. The Quarterly Journal of Experimental Psychology.

[27] Barnes KA, Howard JH, Howard DV, Gilotty L, Kenworthy L (2008). Intact implicit learning of spatial context and temporal sequences in childhood autism spectrum disorder.. Neuropsychology.

[28] Remillard G (2008). Implicit learning of second-, third-, and fourth-order adjacent and nonadjacent sequential dependencies.. The Quarterly Journal of Experimental Psychology.

[29] Schvaneveldt R, Gomez R (1998). Attention and probabilistic sequence learning.. Psychological Research.

[30] Toichi M, Kamio Y (2003). Long-Term Memory in High-Functioning Autism: Controversy on Episodic Memory in Autism Reconsidered.. Journal of Autism and Developmental Disorders.

[31] Ben Shalom D (2003). Memory in autism: review and synthesis.. Cortex.

[32] Minshew NJ, Goldstein G (2001). The pattern of intact and impaired memory functions in autism.. Journal of Child Psychology and Psychiatry.

[33] Krakauer JW, Shadmehr R (2006). Consolidation of motor memory.. Trends Neurosci.

[34] Lord C, Risi S, Lambrecht L, Cook EHJ, L. LB (2000). The autism diagnostic observation schedule-generic: a standard measure of social and communication deficits associated with the spectrum of autism.. Journal of Autism and Developmental Disorders.

[35] Lord C, Rutter M, Le Couteur A (1994). Autism Diagnostic Interview-Revised: a revised version of a diagnostic interview for caregivers of individuals with possible pervasive developmental disorders.. Journal of Autism and Developmental Disorders.

[36] Nemeth D, Janacsek K, Londe Z, Ullman MT, Howard D (2010). Sleep has no critical role in implicit motor sequence learning in young and old adults.. Experimental Brain Research.

[37] Song S, Howard JH, Howard DV (2007). Sleep does not benefit probabilistic motor sequence learning.. Journal of Neuroscience.

[38] Howard DV, Howard JH, Japikse K, DiYanni C, Thompson A (2004). Implicit sequence learning: effects of level of structure, adult age, and extended practice.. Psychol Aging.

[39] Nemeth D, Hallgato E, Janacsek K, Sandor T, Londe Z (2009). Perceptual and motor factors of implicit skill learning.. Neuroreport.

[40] Happé F (1995). The role of age and verbal ability in the theory of mind task performance of subjects with autism.. Child Development.

[41] Hill E, Berthoz S, Frith U (2004). Brief report: cognitive processing of own emotions in individuals with autistic spectrum disorder and in their relatives.. Journal of Autism and Developmental Disorders.

[42] Happé F, Frith U (2006). The Weak Coherence Account: Detail-focused Cognitive Style in Autism Spectrum Disorders.. J Autism Dev Disord.

[43] Song S (2009). Consciousness and the consolidation of motor learning.. Behavioural Brain Research.

[44] Robertson EM, Pascual-Leone A, Press DZ (2004). Awareness modifies the skill-learning benefits of sleep.. Current Biology.

[45] Howard DV, Howard JH, Japikse K, DiYanni C, Thompson A (2004). Implicit sequence learning: effects of level of structure, adult age, and extended practice.. Psychology and Aging.

